# Editorial: The Role of Natural Products in Chronic Inflammation

**DOI:** 10.3389/fphar.2022.901538

**Published:** 2022-04-29

**Authors:** Wenjun Deng, Hongzhi Du, Dahui Liu, Zhaocheng Ma

**Affiliations:** ^1^ Key Laboratory of Horticultural Plant Biology (Ministry of Education), College of Horticulture and Forestry, Huazhong Agricultural University, Wuhan, China; ^2^ College of Pharmacy, Hubei University of Traditional Chinese Medicine, Wuhan, China

**Keywords:** natural products, chronic inflammation, inflammation-related diseases, anti-inflammatory, target, signaling pathway

Inflammation including acute and chronic inflammation is the body’s defense response to external stimuli, such as pathogens, irritants, or infections. Inflammation is usually beneficial to the body. However, repeated stimulation of harmful substances or ineffective regulation of acute inflammation can lead to chronic inflammation (Ma et al., 2021). Persistent inflammatory responses may lead to chronic inflammation-related diseases such as cardiovascular, neurodegenerative, and inflammatory bowel disease (Hussain et al., 2020). In addition, chronic inflammation is also involved in the physiological and pathological processes of various diseases, such as atherosclerosis (AS), rheumatoid arthritis (RA), depression, obesity, gout, Alzheimer’s disease, and cancer (Bai et al., 2021). Currently, steroidal and non-steroidal anti-inflammatory drugs (NSAIDs) with anti-inflammatory, analgesic and other curative effects, are the most commonly used classical treatments for inflammation in clinical practice. However, long-term use of these drugs can cause kinds of adverse reactions (Borquaye et al., 2017), such as gastrointestinal damages (gastric ulcers, bleeding, etc.), liver and kidney dysfunction, and skin diseases (Bai et al., 2021). While rational drug use, drugs with higher safety should be used as much as possible. Therefore, finding natural plant compounds without toxic side effects and good curative effects to replace traditional anti-inflammatory drugs is an urgent issue to be solved in the clinical treatment of inflammation-related diseases.

Natural products have more pharmacological activities and low toxicity properties, which can serve as potential resources for the development of natural anti-inflammatory drugs. Although some severe symptoms of chronic conditions are difficult to be treat with natural products, which are more effective for relieving light pain in the early stage of these disease. Current studies have found that natural products with anti-inflammatory activity include polysaccharides, flavonoids, polyphenols, alkaloids, terpenes, natural pigments, plant volatile oils, quinones, and other compounds (Wu et al., 2021). In recent years, great progress has been made in the development and resolution mechanisms of chronic inflammation-related diseases, and the use of natural products to alleviate inflammatory diseases. We are committed to exploring the anti-inflammatory mechanism and action targets of natural anti-inflammatory components in the context of chronic inflammation, establishing a rapid screening and identification method of natural products, and a technical system for mining action targets, to develop natural products with strong anti-inflammatory activity and no side effects, that can replace traditional anti-inflammatory drugs.

In this Research Topic, the role of natural polysaccharides, alkaloids, terpenes polyphenols in inflammation-related diseases are discussed. Wang et al. reported for the first time the role of *Bacteroides fragilis* polysaccharide A (PSA) in the LPS-induced abnormal hepatic metabolism of voriconazole (VRC). They showed that PSA improved the abnormal metabolism of VRC by inhibiting the TLR4-mediated NF-κB signaling pathway, and they also discussed the possibility of PSA as a clinical adjuvant therapy. Yin et al. found that lentinan, a plant polysaccharide extracted from *Lentinus edodes*, exerted an anti-inflammatory effect by inhibiting the activation of the Wnt/β-catenin pathway. Flavonoids are a major anti-inflammatory natural product that is widely used in the treatment of various chronic inflammatory diseases. Wu et al.found that the anti-inflammatory effect of hesperetin could effectively prevent cartilage degradation, suggesting that hesperetin may be a potential natural product for the treatment of osteoarthritis (OA). Alkaloids are one of the most important functional compounds in Chinese herbal medicine with significant biological activity. Yu et al. found that tomatidine with anti-inflammatory, antitumor and immunomodulatory activities, inhibited the destructive behaviors of fibroblast-like synoviocytes and ameliorated RA in rats. Another interesting study performed by Wang et al.analyzed the effect of evodiamine (EVO) on inflammation and cancer from the perspective of colitis-associated cancer (CAC), and they found that EVO may be a new drug to prevent CAC through regulating gut microbiota metabolites. Terpenes including monoterpenes, sesquiterpenes, diterpenoids, etc., are major sources for the study of natural products and the development of new drugs. Betulin, a natural triterpene extracted from birch bark, could be used to treat OA. Ren et al. found that betulin can ameliorate OA by reducing inflammation through the AKT/Nrf2/HO-1/NF-κB signaling pathway. In addition, Ma et al. described the sesquiterpene compound bilobalide as a unique component in *Ginkgo biloba* L. extract with strong anti-inflammatory activity. From their findings, bilobalide was found to be a potential natural therapeutic drug for OA. Polyphenols are widely found in plant foods and have antioxidant and anti-inflammatory activities. Yao et al. identified HDAC11 protein as the target of the natural polyphenolic compound hydroxytyrosol acetate (HF-AC) in olive oil through molecular docking and drug affinity responsive target stability. This study provided a new insight into chronic inflammation-induced atherosclerosis treatment strategy.

Additionally, some medicinal plant extracts and classic traditional Chinese medicine formulas are also used for the development of anti-inflammatory drugs. Medicinal plant extracts have complex components and high medicinal value. Feng et al. used the screening method of multi-target affinity ultrafiltration combined with HPLC-MS to quickly screen out the multi-target bioactive components from *Podophyllum sinense*, which provided an effective method for mining new multi-target drugs from natural products. In terms of natural extracts, Balgoon et al. compared the effects of topical and oral administration of pumpkin extract (PE) with betamethasone-treated groups and found that PE could be used as an alternative or supplementary method for the treatment of contact dermatitis associated with depression. Classic traditional Chinese medicine formulas are another effective method for the clinical treatment of chronic diseases. Xu et al. explored the role of Huangbai liniment (HB) in the treatment of skin inflammation in Atopic dermatitis (AD). The results showed that HB can significantly reduce the expression of pro-inflammatory factors and effectively relieve skin inflammation in AD patients by restoring the T cell immune balance. It provided a research basis for HB to develop new AD treatments in the future. Yue et al. explored the therapeutic effect of Shu Gan He Wei decoction (SHD) on depression, anxiety and cecal mucosal injury in chronic stress model rats. It provided a new therapeutic method for the clinical treatment of TCM regulation of inflammation-related diseases. Also, Xia et al.studied the effect of Yi Shen Juan Bi pill on the homeostasis of the bone immune microenvironment in RA.

Natural products are critical in new drug development due to their broad chemical and functional diversity. The authors of this Research Topic have tried to establish a system for rapid screening and identification of bioactive components and looked for natural products that can replace NASIDs. In summary, the articles in this Research Topic provide new discoveries that natural products can be used to treat chronic inflammation-related diseases and offer useful information for the development of natural products and their derivatives. To expand the wide application of natural products in the treatment of chronic inflammatory diseases, it is necessary to further explore bioactive components with multiple targets and multiple pharmacological activities. And it needs to improve the purity and effectiveness of natural products through separation technology. However, the development of natural products is currently only at the *in vitro* experimental stage, and randomized clinical trials are needed to evaluate the safety and efficacy of natural products for inflammation-related diseases under specific conditions. More importantly, how to improve the safety and efficacy of natural products in the treatment of diseases.
